# Epidemiology, Diagnosis, and Risk Factors of *Helicobacter pylori* Infection in Children

**DOI:** 10.5005/jp-journals-10018-1208

**Published:** 2017-05-05

**Authors:** Gokben Ozbey, Alfizah Hanafiah

**Affiliations:** 1 Department of Medical Laboratory, Firat University, Elazig, Turkey; 2 Department of Medical Microbiology and Immunology Faculty of Medicine, Universiti Kebangsaan Malaysia, Cheras, Kuala Lumpur, Malaysia

**Keywords:** Children, Diagnostic tests, Epidemiology, *Helicobacter pylori.*

## Abstract

*H. pylori* infection is a global public health problem associated with some gastrointestinal diseases in children, especially in developing countries, since prevalence of *H. pylori* is low in the developed world. Both noninvasive (stool antigen test, urea breath test, and blood test) and invasive (histology, rapid urease test, and microbiological culture) tests have been utilized to detect *H. pylori* infection. However, a single test is not reliable enough and does not provide accurate enough data to determine *H. pylori* infection among children. Risk factors of *H. pylori* infection in children were related to ethnicities, household properties, geographic location, living conditions, water sources, type of housing, presence/absence of sewage systems, and garbage collection within the living environment. These risk factors were usually associated with the socioeconomic status of the family. This review article aims to determine the gaps in the knowledge of the epidemiology, risk factors, and diagnostic tests of *H. pylori* infection among children.

**How to cite this article:** Ozbey G, Hanafiah A. Epidemiology, Diagnosis, and Risk Factors of *Helicobacter pylori* Infection in Children. Euroasian J Hepato-Gastroenterol 2017;7(1):34-39.

## INTRODUCTION

*H. pylori,* which was discovered in 1982, is the most frequently occurring chronic bacterial infection in developing countries.^1^ The prevalence of the disease is high - 90% in developing countries - whereas the prevalence is less than 40% in developed countries excluding Japan.^2,3^

Techniques utilized to detect *H. pylori* infection are grouped as invasive and noninvasive tests and include the rapid urease test (RUT), microbiological culture, histology, and polymerase chain reaction (PCR), in which esophagogastroduodenoscopy (OGDS) is required to obtain the stomach biopsy.^1,4^ Noninvasive methods consist of the stool antigen test, urea breath test (UBT), and blood test for detection of *H. pylori* antigens or anti-*H. pylori* antibody.^5^ However, a single test is not reliable enough and does not provide accurate enough data to be used as a gold standard to determine *H. pylori* infection among children.^1^ Accordingly, it is suggested to obtain correlative results of at least two tests to determine the *H. pylori* infection in children.^1,6^ Endoscopy cannot clinically specify and is not applicable in many studies to diagnose *H. pylori* infection in children.^7^

## EPIDEMIOLOGY

More than half of the world’s entire population is known to be infected with *H. pylori,* it is generally acquired during the first 5 years of life.^8,9^ The proportion of infection of *H. pylori* acquired by children ranges from 30 to 50%, whereas it reaches a limit of over 90% during adulthood in developing countries.^10^ In developed countries, the prevalence of the infection in children is low (1.2-12.2%) compared with developing countries where *H. pylori* is the most frequently isolated bacteria in a 10-year-old.^11-14^ This may be explained by the poor hygiene and sanitation, low socioeconomic status, and overcrowded conditions, which increase the risk of infection.^15^

Infection is rare among young children and accounts for up to 60% in older people in developed countries.^16-18^ The prevalence of *H. pylori* infection in children differs among nation and regions in the same country. The prevalence of infection has suggested a significant decline in many parts of developed countries, such as Western Europe and North America.^18^ However, such a decrease has not been reported in developing countries, such as eastern and southern parts of Europe and Asia.^18^

In developing countries, the prevalence of *H. pylori* infection may differ among countries, in which the highest prevalence was detected among the Bangladeshi children (80%) followed by the Indians (57%).^19,20^ The *H. pylori* infections among children in Brazil and Mexico, countries which are located in Latin America, have similar prevalence rates.^21,22^ Similar *H. pylori* prevalence rates have also observed among children in Egypt and Saudi Arabia.^23,24^ However, Vietnam showed the lowest prevalence rate of *H. pylori* infection, which might be due to the age of children included in the study, the youngest (less than 3 years old) when compared with the other countries.^25^ In Turkey, 41% of *H. pylori* prevalence rate was detected among children aged less than 6 years.^26,27^

A prospective longitudinal cohort study (Gambian children aged 3 months to 2 years) was performed at intervals of 3 months for 2 years.^28^ The prevalence increased ranging from 19% at 3 months of age to 84% by 30 months of age.^28^ In developed countries, in a cohort of Swedish children who were followed from the ages of 6 months to 11 years, *H. pylori* infection was observed in 13.6% of children aged 18 and 24 months.^29^ Whereas, at age 11 years, only 3% of the children were seropositive.^29^ In other studies, the prevalence rates were found to be 8.6 and 2.4% among the Irish and German children aged 3 years respectively.^30,31^ Okuda et al^32^ found a prevalence proportion of 3.7% in Japanese children aged less than 2 years.^32^

Studies from Turkey showed an overall prevalence of *H. pylori* of 78.5% in children aged from 7 to 14 years in 1990, 66.3% in 2000,^33^ and 30.9% among those from 2 to 12 years in 2008.^34^ Ozbey et al^35^ detected that the prevalence of *H. pylori* infection in the period from March 2011 to September 2012 in eastern Turkey was 66.3% in children from age 4 to 18 years.^35^ In addition, another study showed no association between severe clinical diseases and genotypes in children.^36^

## RISK FACTORS FOR INFECTION

Earlier studies reported that risk of *H. pylori* infection can change depending on race/ethnicity, household properties, and geographic location.^37^ Table 1 shows the relation of various risk factors with *H. pylori* infection in various regions.

Socioeconomic status is a major risk factor in the acquisition of *H. pylori* infection.^34^ The infection rates were high in a low socioeconomic group, followed by a middle-level group, and the lowest in a high-level group.^34,38,39^ This relationship was obvious in developing countries; however, in developed countries, the risk factor did not have a significant role in acquisition of *H. pylori.^40,41^*

Many studies showed that the education level of the parents and increased numbers of siblings were the major factors influencing the *H. pylori* positivity in children,^34,39,42-44^ while the others showed no asso-ciation.^40,41,45^ Other risk factors, such as water sources, typing of housing, presence/absence of sewage system, and garbage collection were related with the living conditions and usually associated with the socioeconomic status of the family.^46^

This indicated that the risk factors in acquisition of *H. pylori* infection in children vary among different populations and reflect the exposure which occured during the early years of life.

## DIAGNOSTIC TECHNIQUES OF *H. PYLORI* INFECTION

### Invasive Tests

Histology

A routine hematoxylin and eosin (H&E) stain detects *H. pylori* and inflammation (gastritis type).^8^ When this stain has produced inconclusive results, special stains, such as Giemsa, Warthin-Starry, acridine orange, tolu-idine blue, Dieterle, Genta, Romanouski and McMullen stains, or immunochemical methods can be utilized.^2^ The Genta stain, which combines silver, H&E, and Alcian blue stains, is used to observe both inflammatory cells and *H. pylori,* but it is expensive and technically complex.^2^ However, the Giemsa stain is much easier to perform, highly sensitive, inexpensive, and the preferred method for clinical practice.^2^ The other methods can be utilized for research proposes.^2,47^

The main advantage of histologic testing is a direct visualization of pathologic changes related to *H. pylori* infection including severity of inflammation, grade of atrophy, the development of intestinal metaplasia, and malignancy.^48,49^

Histologic testing has a few disadvantages: The use of some medications, such as antibiotics, bismuth, and proton pump inhibitors (PPIs) decreases the sensitivity and specificity of the test.^48^ The cost of the special stains is quite high, and skilled personnel are needed to examine the slides.^48^ Histopathologic studies are less often used to diagnose *H. pylori* infection among children since OGDS is required.^2^

**Table 1:** **Table 1:** Association of risk factors with *H. pylori* infection in different regions

						*Association of risk factors with* H. pylori *infection*		*References*			
*Country*		*Study population (number of subjects)*		*Age range (years)*		Yes		No			
Czech		Healthy children		0-15		• Two or more children in the household				Sykora et al^42^	
Republic		(1,545)		0-15							
						• Institutionalization of the child					
						• Lack of formal education of the father					
Greece		Symptomatic		Mean				• Socioeconomic status		Roma et al^41^	
		children (100)		11.02				• Parental educational level			
								• Number of children in the household			
								• Sharing a room or a bed with parents or siblings			
Taiwan		Healthy high-school		Mean 14.3				• Number of siblings		Chi et al^40^	
		students (106)						• Household size			
								• Parental educational level			
								• Family income			
Pakistan		Children (1,976)		1-15		• Lower socioeconomic status		• Water source to household		Jafri et al^39^	
						• Lower educational status of the child’s father		• Tyoe of housing		Jafri et al^39^	
Turkey		Healthy children		2-12		• Lower family income				Yucel et al^34^	
		(165)				• Poor living conditions					
						• Lower educational status of the mother					
						• Higher number of siblings					
Italy		Healthy children		5-16		• Living in rural areas		• Socioeconomic status for children in rural areas		Fujimura et al^38^	
		(2,810)				• Contact with dogs for children in rural areas		• Breast-fed history for children in rural and urban areas			
						• Lower socioeconomic status for children in urban areas					
						• Attending daycare centers for children in urban areas					
Sao		Healthy children		Mean 6.82				• Gender		Miranda et al^45^	
Paulo,		(326)						• Race (white or nonwhite)			
Brazil								• Breast-feeding			
								• Number of people in the home			
								• Number of rooms			
								• Bed sharing			
								• Living in a shantytown			
								• Family income			
								• Nutritional status			
Brazil		Healthy children		0.5-12		• Absence of a sewage system		• Maternal educational level		Parente et al^46^	
		(303)				• Absence of garbage collection service					
						• Absence of indoor plumbing					
Brazil		Healthy children		Mean 6.1		• Increased number of children in the household				Queiroz et al^44^	
		(133)				• Use of well water					
Brazil		Healthy children		Mean 6.8		• A larger sibling number				Dattoli et al^43^	
		(1,104)				• Nursery attendance					
						• Location of the house at an unpaved street					
						• Absence of a flush toilet					

Rapid Urease Test

The test uses the ability of the organism to produce large quantities of urease enzyme for diagnosis of the infection in tissue biopsy specimens.^2,8^ Being rapid, inexpensive, commonly available, and highly specific are the advantages of RUT as a preferable diagnostic test to be used for detecting *H. pylori* infection.^2,50^

Some members of the microbiota in the oropharynx make urease; however, this weaker enzyme is destroyed rapidly when it reaches the stomach due to the high acidity of the gastric juice.^2^ In addition, a recent intake of bismuth compounds, antibiotics, PPIs and patients with achlorhydria can result in false-negative urease test.^2^

Culture

Culture that requires an endoscopy is the gold standard and the most specific method for diagnosing *H. pylori.^2^*It is used for determining antibiotic susceptibility of *H. pylori* in clinical practice.^2^ However, the results vary according to the microbiologist’s experience, transport media, and specimen quality used.^2^ In addition, the culture is relatively difficult to perform, expensive, time-consuming, and needs special media.^8^

Molecular Methods

The PCR tests and other molecular methods have been reported to be the most reliable and accurate methods to detect *H. pylori.^51,52^* The PCR can be performed rapidly and cost-effectively, used to detect different bacterial genotypes, and employed in pathogenic and epidemiological studies.^2^ The PCR can be carried out on tissue and stool specimens and helps identify genes related to antibiotic resistance and virulence.^4^ Kalach et al^53^ described a quantitative real-time PCR (qPCR) used to detect *H. pylori* in gastric biopsy samples of French children.^53^ They reported that qPCR is a more sensitive test than histology, culture, or RUT alone, and allows detecting low bacterial loads.^53^

Fluorescent *in situ* hybridization (FISH) is a recently developed technique, which is used to detect the resistance of *H. pylori* to clarithromycin.^2^ The advantage of FISH is that simultaneous detection of *H. pylori* and mac-rolide resistance can be done on the same formalin-fixed paraffin-embedded gastric biopsies used for histologi-cal assessment.^2,54^ However, conventional methods for antibiotic susceptibility testing, such as E-test and agar dilution method are dependent on bacterial growth.^54^ In addition, the test is expensive, labor intensive, and not widely used in clinical studies.^2^

Omics-based methods are increasingly being used for diagnosis of *H. pylori* infection.^55^ Authors detected *H. pylori* by pyrosequencing method of the 16S ribosomal ribonucleic acid gene in all samples that were detected to be *H. pylori* positive by conventional methods and in 60% of the *H. pylori*-negative samples.^56^

### Noninvasive Tests

Serology

Serological tests are qualitative, commonly used to detect immunoglobulin (Ig)G, IgA, or IgM antibodies to *H. pylori* infection, and are accepted as first-line nonin-vasive diagnostic methods among adults with suspected *H. pylori* infection in Europe.^5^ However, serology does not indicate whether or not the infection is active or past.^5^ In general, no serological assays can be utilized on their own in adolescents and children for diagnosing *H. pylori* infection. They cannot be used to observe the success of eradication therapy since the sensitivity and specificity for determination of antibodies (IgG or IgA) to *H. pylori* in children differ commonly.^4^ A positive IgG test can result several months or even years after the infection, and is not reliable for diagnosis or treatment outcomes.^4^

Urea Breath Test (UBT)

The UBT is a reliable and noninvasive technique and widely used for determining of *H. pylori* infection in adults and to confirm or monitor the eradication therapy.^1,5^ However, it has less accuracy for the detection of *H. pylori* infection in infants and young children.^1,5^ The [^13^C] UBT is the best detection test in children aged 5 years and older and may be accepted as a "gold standard," especially if endoscopy is not routinely performed.^5^ Due to radioactivity risks in children, a few reports are available on the use of ^14^C-labeled UBT in children.^47^ False-negative results can occur in patients who have recently received bismuth compounds, antibiotic agents, or gastric acid antisecretory agents.^5^

### Stool Antigen Test

Although *H. pylori* stool antigen test is an excellent technique, compared with other techniques,^1,57^ its sensitivity and specificity depend on types of commercial test used, treatment status, cut-off value, and the interpretation of weakly positive results.^6,57^

## CONCLUSION

In developing countries, failures in appropriate diagnosis and treatment are the result of deficiencies in health systems and socioeconomic factors. It is necessary to carry out future studies to develop new technologies, including diagnostic tests for detection of *H. pylori* infection among children, which will be useful in developing countries, where the prevalence rate of infection is common. Detection of early infection among children may reduce the burden of peptic ulcer and gastric cancer later in life. This will further reduce the cost for management and treatment of the *H. pylori*-associated diseases.
